# Clinical relevance of low bone density in cystic fibrosis adult patients: A pilot study

**DOI:** 10.1097/MD.0000000000032227

**Published:** 2023-01-06

**Authors:** Sandra Dury, Julien Ancel, Bruno Ravoninjatovo, Isabelle Lambrecht, Jeanne-Marie Perotin, Pauline Mulette, François Lebargy, Jean-Hugues Salmon, Gaëtan Deslée, Claire Launois

**Affiliations:** a Department of Respiratory Diseases, Reims University Hospital; b EA7509 IRMAIC, University of Reims Champagne-Ardenne; c Department of Rheumatology, Reims University Hospital; d INSERM UMRS 1250, Reims University Hospital, Reims.

**Keywords:** adult, bone mineral density, cystic fibrosis, disability, pain, quality of life

## Abstract

Survival improvement in cystic fibrosis (CF) is associated with more frequent long-term complications, including CF related bone disease (CFBD). Impact of CFBD on global health outcome remains poorly described. We aimed to assess the relationship between low bone mineral density (BMD) and spinal pain, disability, and quality of life in CF adult patients. This monocentric cross-sectional study with prospective data collection was conducted from November 2016 to December 2019 in the Department of Respiratory Diseases at the University Hospital of Reims (NCT02924818). BMD was assessed by X-ray absorptiometry (DXA). Disability was assessed by the Health Assessment Questionnaire (HAQ). Quality of life was assessed by both the St George’s Respiratory Questionnaire and the Cystic Fibrosis Questionnaire for teenagers and adults (CFQ 14+). Forty patients were analyzed, 68% of men, with a median age of 25 years, a median body mass index of 21 kg/m² and a median FEV_1_% of 54%. Nine patients (23%) had spinal pain. Ten patients (25%) had a low BMD. Compared with patients with normal BMD, patients with low BMD had a significantly lower BMI (22 vs 19 kg/m²; *P* = .006) and less vitamin D supplementation (33% vs 0%; *P* = .035). Low BMD was not associated with spinal pain, disability and quality of life. Low BMD is frequent in CF, affecting 1-quarter of adult patients. No significant association was found between low BMD and spinal pain, disability or quality of life.

## 1. Introduction

Cystic fibrosis (CF) is the most common life-threatening genetic disease in Caucasian populations. With survival improvement, the prevalence of long-term complications including rheumatologic disorders increased.^[1]^ CF related bone disease (CFBD) defined as low bone mineral density (BMD) is frequently encountered in adult patients (13%–34%). Many factors including CFTR dysfunction, calcium deficiency, malnutrition and CF related diabetes contribute to its development and severity.^[2]^ Low BMD increases the risk of rib and spinal fractures.^[3]^To date, the clinical impact of CFBD on quality of life has not been investigated.

Our study aimed to assess the relationships between BMD and spinal pain, patient’s disability and quality of life.

## 2. Methods

### 2.1. Study design

This monocentric cross-sectional study with prospective data collection was conducted from November 2016 to December 2019 in the Department of Respiratory Diseases at the University Hospital of Reims. All CF patients were considered for inclusion and included in the observational cohort of chronic inflammatory lung diseases, the RINNOPARI study (Recherche et INNOvation en PAthologie Respiratoire Inflammatoire) (NCT02924818). The study was approved by the Ethics Committee of Dijon EST I on May 31st, 2016 (N°2016-A00242-49) and by the French National Agency for Medicines and Health Products (ANSM) on April 25th, 2016. Each patient signed a written informed consent form.

Patients were included in the current analysis if they were at least 18 years of age and had undergone a dual X-ray absorptiometry (DXA) within 6 months at inclusion in the RINNOPARI cohort. Patients were excluded if they required an urgent visit or if they had a previous or planned lung transplantation. Forty consecutive patients fulfilled the inclusion/exclusion criteria.

### 2.2. Rheumatologic assessment

Rheumatologic pain and arthritis were assessed using a questionnaire as previously described.^[4]^ For this study, the question regarding spinal pain (“Did you have spinal pain?”) was considered.

A baseline DXA assessment is recommended in CF adult patients with a routine monitoring.^[5]^ According to the European guidelines for CF, we used the Z-score for premenopausal women and men under the age of 50 years. DXA was measured at lumbar spine (L1–L4), femoral neck and upper femoral extremity. Low BMD was defined by at least 1 Z-score below −2.0 standard deviations.^[6]^

### 2.3. Disability score and quality of life scales

The Health Assessment Questionnaire (HAQ) was used to assess patients’ abilities in the past week. This scale includes 8 sections: dressing, arising, eating, walking, hygiene, reach, grip and activities.^[7]^

Quality of life was evaluated by both the Cystic Fibrosis Questionnaire for teenagers and adults (CFQ 14+)^[8]^ and the St George’s Respiratory Questionnaire.^[9]^

### 2.4. Data analysis

Data were analyzed as numbers (percentages), median and range [1st–3rd quartiles]. There was not sample size calculation in this pilot study. Fisher’s exact tests were used for qualitative variables and Mann–Whitney *U* tests were used for quantitative variables. A *P* value < .05 was considered as significant. Results were analyzed with SPSSv27. The datasets analyzed during the current study are available from the corresponding author on reasonable request.

## 3. Results

### 3.1. Patient characteristics

Patients were mostly men (68%). Median age was 25 years. Median body mass index (BMI) was 21 kg/m². Median FEV_1_% was 54%. Treatment included inhaled (n = 17, 43%) and oral (n = 1, 3%) glucocorticoids (Table 1).

**Table 1:** Clinical, functional and biological characteristics according to low BMD.

	Total	Low BMD (Z *<* −2)	Normal BDM (Z > −2)	*P* value
n (%)	40	10 (25)	30 (75)	
Male	27 (68)	8 (80)	19 (63)	ns
Age, yrs	25 [21;33]	22 [20;30]	26 [21;34]	ns
BMI, kg/m²	21 [19;23]	19 [18;20]	22 [20;24]	.006
CFTR mutation				ns
ΔF508 homozygous	15 (38)	4 (40)	11 (37)	
ΔF508 heterozygous	22 (55)	5 (50)	17 (57)	
Other	3 (8)	1 (10)	2 (7)	
Exocrine pancreatic insufficiency	31 (78)	9 (90)	22 (73)	ns
Diabetes	12 (30)	2 (20)	10 (33)	ns
Regular physical activity	28 (70)	9 (90)	19 (63)	ns
FEV_1_, % predicted	54 [37;93]	43 [36;79]	55 [37;94]	ns
Respiratory exacerbation in the past year	10 (25)	1 (10)	9 (30)	ns
Biochemical serum level (n = 35)				
Calcium (mmol/L)	2.2 [2.2;2.3]	2.3 [2.2;2.3]	2.2 [2.2;2.3]	ns
25OH Vitamin D (ng/mL)	27 [17;33]	31 [23;34]	26 [15;32]	ns
Glucocorticoid use				
Long term inhaled	17 (43)	5 (50)	12 (40)	ns
Oral steroid use in the previous year	1 (3)	1 (10)	0 (0)	ns
Vitamin D supplementation	10 (25)	0 (0)	10 (33)	.035
Bisphosphonate use	1 (3)	1 (10)	0 (0)	ns

Data are expressed as frequency (percentage) or median and range [1st–3rd quartiles].

BMD = bone mineral density, BMI = body mass index, CFTR = cystic fibrosis transmembrane regulator, FEV_1_ = forced expiratory volume, ns = non-significant.

### 3.2. Rheumatological assessment

Nine patients (23%) had spinal pain.

Median Z-score was −0.8 [−1.7; −0.27] on lumbar spine, −0.55 [−1.45; −0.17] on femoral neck and −0.75 [−1.32; −0.1] on upper femoral extremity. Ten patients (25%) had a low BMD.

Patients with low BMD had a significantly lower BMI than normal BMD patients (19 vs 22 kg/m²; *P* = .006). Vitamin D supplementation was used in 10 patients (33%) with normal BMD and in no patient with low BMD (*P* = .035). There was no difference regarding CFTR mutation, diabetes, glucocorticoid use, serum calcium levels, respiratory exacerbations in the last year and regular physical activity (Table 1).

### 3.3. Relationships between low BMD and spinal pain, disability and quality of life scales

Low BMD was not associated with spinal pain, HAQ and quality of life assessed by both the St George’s respiratory questionnaire and CFQ 14 + scores (Table 2).

**Table 2:** Relationship between low BMD and spinal pain, disability and quality of life.

	Total	Low BMD (Z *<* −2)	No low BMD (Z > −2)	*P* value
n (%)	40	10 (25)	30 (75)	
Spinal pain	9 (23)	3 (30)	6 (20)	ns
HAQ†	0 [0;0.25]	0 [0;0]	0 [0;0.25]	ns
SGRQ¶				
Impact	19 [8;28]	18 [4;33]	19 [12;26]	ns
Activity	33 [17;59]	21 [16;43]	35 [22;60]	ns
Symptoms	45 [36;63]	50 [30;61]	44 [36;63]	ns
Total	25 [13;41]	15 [11;31]	27 [16;42]	ns
CFQ 14+#				
Physical functioning	75 [48;85]	75 [50;83]	71 [48;86]	ns
Role perception	83 [67;92]	75 [54;92]	83 [67;92]	ns
Vitality	50 [42;67]	58 [50;67]	50 [42;75]	ns
Emotion	80 [60;93]	73 [53;93]	80 [60;93]	ns
Social Perception	61 [50;72]	61 [56;72]	61 [50;68]	ns
Body image	67 [47;78]	67 [33;67]	67 [56;78]	ns
Eating disturbance	100 [89;100]	100 [67;100]	100 [89;100]	ns
Treatment burden	56 [44;67]	56 [44;67]	61 [47;75]	ns
Health perception	56 [36;67]	56 [33;56]	67 [44;67]	ns
Weight	67 [33;100]	67 [33;67]	67 [33;100]	ns
Respiratory symptoms	67 [62;71]	64 [62;74]	71 [57;71]	ns
Digestive symptoms	83 [78;100]	78 [67;89]	89 [78;100]	ns

Data are expressed as frequency (percentage) or median and range [1st–3rd quartiles].

CFQ 14 + = cystic fibrosis questionnaire for teenagers and adults, HAQ = health assessment questionnaire, ns = non-significant, SGRQ = St George’s Respiratory Questionnaire.

† The final score ranged between 0 (no assistance) and 3 (patient needs special device and help from another person).^[6]^

¶ A score of 100 indicates maximal impairment of quality of life.^[8]^

# The score ranges from 0 to 100, the highest score corresponding to a better quality of life.^[7]^

## 4. Discussion

So far few studies have investigated the clinical relevance of CFBD. For the first time, we analyzed in CF adult patients the relationships between low BMD and spinal pain, disability and quality of life. We confirmed that low BMD is frequent (25%) but we showed no clinical impact on physical health in this cross-sectional study.

As previously reported^[10,11]^ we found a significant association between BMD and BMI. CF adult patients exhibit a more frequent decrease in BMD than non-CF age-matched controls.^[12]^ Besides low BMI, CFTR dysfunction, calcium deficiency, malnutrition and diabetes are also involved in the development of low BMD in CF.^[2]^

Of notes, the clinical relevance of low BMD in CF is not clearly established. Prevalence of vertebral fractures in CF adults ranges from 7.2% to 26.7%.^[13,14]^ In late-stage of the disease, rib fractures and vertebral compression are 10- and 100-fold more frequent in CF than in the general population, respectively.^[15]^ CF patients with lower muscle strength have significantly worse BMD.^[16]^ The association between low BMD and airway clearance impairment and/or decline of respiratory function has not been demonstrated.^[11,13,17,18]^ Some authors consider that osteoporosis may be a barrier to lung transplantation.^[19,20]^

Interestingly, we found no association between spinal pain and low BMD in our study. In general population, osteoporosis - called the “silent thief” - often causes few symptoms for a long time until the occurrence of pathologic fractures, a well-known cause of acute and chronic pain.^[21]^ No previous study specifically addressed the relationship between rheumatologic pain and CFBD. Many rheumatologic disorders related to CF have been described to explain spinal pain, including vertebral deformity, scoliosis, rib and vertebral fractures.^[22]^

Our study showed no relationships between low BMD, disability and quality of life. The impact of CF rheumatologic disorders on functional disability and health-related quality of life has not been fully investigated.^[23]^ A reduced mobility of the spine, a higher intensity of spinal pain and a more marked impairment of HAQ has been found in CF adult patients.^[12]^ We recently showed a more important impairment on HAQ scale in patients with both spinal and joint pain.^[4]^ CF quality of life is associated with several factors including FEV_1_%, BMI, pulmonary exacerbations, and depression and anxiety.^[23,24]^

Several limitations of our study have to be highlighted. First, our study is monocentric with a small sample size, then limiting the possibility to identify clinically relevant differences between patients with or without low BMD. Second, all risk factors for low BMD were not available including delayed puberty and systemic inflammation. Third, complications including non-vertebral and vertebral fractures were not investigated. The prevalence of osteoporosis defined in young adults by having a Z-score below −2 and a significant fracture history, could then not be determined in our study.^[6]^ Lastly, the cross-sectional design of our study assessing the clinical relevance of low BMD in a young cohort of CF patients does not allow to assess long term complications and impact on physical health.

## 5. Conclusion

In CF adult patients, our study showed that low BMD was not associated with spinal pain, disability and quality of life. However, these results have to be cautiously interpreted because of the small sample size of this pilot study. DXA remains a useful exam to detect low BMD in CF adults. Additional longitudinal larger studies are needed to better characterize the long-term impact of low BMD and osteoporosis on physical health in CF.

## Author contributions

**Conceptualization:** Sandra Dury, Bruno Ravoninjatovo, Gaëtan Deslée, Claire Launois.

**Formal analysis:** Sandra Dury, Jeanne-Marie Perotin.

**Investigation:** Sandra Dury, Bruno Ravoninjatovo, Isabelle Lambrecht, Pauline Mulette, François Lebargy, Jean-Hugues Salmon, Gaëtan Deslée.

**Visualization:** Sandra Dury.

**Writing – original draft:** Sandra Dury, Gaëtan Deslée, Claire Launois.

**Writing – review & editing:** Sandra Dury, Bruno Ravoninjatovo, Isabelle Lambrecht, Jeanne-Marie Perotin, Pauline Mulette, François Lebargy, Jean-Hugues Salmon, Gaëtan Deslée, Claire Launois.
